# A co-utilization strategy to consume glycerol and monosaccharides by *Rhizopus* strains for fumaric acid production

**DOI:** 10.1186/s13568-018-0601-8

**Published:** 2018-04-30

**Authors:** Sylwia Kowalczyk, Elwira Komoń-Janczara, Agnieszka Glibowska, Adam Kuzdraliński, Tomasz Czernecki, Zdzisław Targoński

**Affiliations:** Department of Biotechnology, Microbiology and Human Nutrition, University of Life Sciences in Lublin, Skromna 8, 20-704 Lublin, Poland

**Keywords:** *Rhizopus*, Fumaric acid, Glycerol, Carbohydrates, Co-fermentation

## Abstract

The ability of *Rhizopus oryzae* to produce fumaric acid in the presence of glycerol and/or various monosaccharides as carbon sources was examined for seventeen different strains of this fungi. These strains were tested in shake-flask cultures on media containing glycerol and seven different carbohydrates, including glucose, fructose, galactose, mannose, xylose, arabinose, and rhamnose. An interesting and applicationally useful phenomenon was observed. This work presents a new approach to the conventional microbiological method of producing fumaric acid. In the presence of 40 g/l glycerol as the sole carbon source, fumaric acid production reached 0.16–6.1 g/l after 192 h. When monosaccharides were used as a single carbon source, the maximum fumaric acid concentration was much higher; for example, 19.8 g/l was achieved when 40 g/l xylose was used. In the co-fermentation of xylose (40 g/l) and glycerol (20 g/l), post-culture broth contained approx. 28.0 g/l of fumaric acid with a process yield of 0.90 g/g after 168 h. The production of fumaric acid by *Rhizopus oryzae* was also increased in the dual presence of glycerol and monosaccharides like fructose, galactose, and mannose. However, results obtained on glucose-glycerol-based medium did not follow this trend, showing instead complete utilization of glucose with significant glycerol consumption, but unexpectedly low final amounts of fumaric acid and process yields. Understanding how *Rhizopus oryzae* utilize various carbon sources may provide alternative avenues of fumaric acid fermentation.

## Introduction

In industrial biotechnology, feedstock is by far the highest cost factor in the production of chemicals, representing 40–60% of total costs (Demain 2007). Therefore, there is an increasing interest in using food waste products, crude glycerol, and lignocellulosic materials as a feedstock for biotechnological processes. Lignocellulosic biomass is composed of three main compounds: cellulose, hemicelluloses, and lignin. Cellulose is a polymer of glucose, and thereby a potential source of fermentable sugar. Hemicellulose hydrolysates consist mainly of xylose, glucose, mannose, arabinose, galactose, traces of other sugars, uronic acid, and acetyl groups, and they offer an attractive possibility to be used as a substrate in fermentation processes. Biodiesel production generates about 10% glycerol as the main byproduct. In recent years, the price of crude glycerol decreased from about 0.25 USD per pound to 0.05 USD per pound. Worldwide, the source of crude glycerol derived from biodiesel conversion increased from 200,000 tons in 2004 to 1,224,000 tons in 2008 (Yang et al. 2012). According to new market research, the global glycerol market is expected to reach 3 billion USD by 2022 (http://www.healthtech.com; http://www.radiantinsights.com). Therefore, it is important to find new applications for crude glycerol. It has been reported that glycerol is a good carbon source for many microorganisms, such as fungi and bacteria involved in the production of value-added fuels and chemicals (da Silva et al. 2009; Nicol et al. 2012).

Fungi of the genus *Rhizopus* are well known producers of important chemicals. A wide range of substrates utilized by these fungi in different processes is a subject of some current reviews (Das et al. 2016; Londoño-Hernández et al. 2017). The production of fumaric acid from lignocellulosic biomass by *Rhizopus* fermentation has received a great deal of attention due to its extensive applications in the polymer, medical, and food industries. As glycerol is a renewable resource, obtaining fumaric acid from glycerol as substrate may become economically attractive. Our preliminary investigations showed that *R. oryzae* produced significantly lower amounts of fumaric acid with crude glycerol as the sole carbon source, when compared with glucose growth cultures (unpublished data). This glycerol utilization phenomenon can be seen from archaebacteria (Falb et al. 2008) to human cells (Hibuse et al. 2006). The fungal glycerol utilization pathway also has already been described in *Rhizopus oryzae* (Huang et al. 2015; Wang et al. 2015). Molecules of glycerol are usually actively transported into fungal cells expending ATP, but facilitated diffusion of glycerol has also been observed (Fakas et al. 2009). Two major pathways of glycerol catabolism under aerobic conditions are known: the phosphorylation pathway (generally found in eukaryotes) and the oxidation pathway. However, there is evidence for some alternative routes. The phosphorylation pathway is based on the action of glycerol kinase, which yields 3-P-glycerol (phosphorylated glycerol). The NAD-linked dehydrogenase then oxidizes it to 3-P-dihydroxyacetone (Courtright 1975). The oxidation route occurs in reverse; first glycerol oxidation by glycerol dehydrogenase yields dihydroxyacetone, then this is phosphorylated to generate 3-P-dihydroxyacetone (Tom et al. 1978). Dihydroxyacetone phosphate derived from either pathway can be shunted to the glycolytic or gluconeogenic pathway and then further transformed (Fakas et al. 2009; Turcotte et al. 2010).

Co-fermentation/co-utilization of two or more substrates is a natural phenomenon that usually occurs when microorganisms have to deal with complex carbon sources. Interactions that occur between individual components after the degradation of large complex molecules and the microorganism’s substrate transport system can affect production efficiency by elevating it or, more frequently, by lowering it. Carbon catabolite repression (CCR) is an important regulatory mechanism utilized by microorganisms, oftentimes appearing as sequential utilization of substrates present in the medium. Microorganisms can quickly adapt to utilization of a readily available and energy-efficient carbon source over relatively less easily accessible carbon sources. In industrial production, the occurrence of such a phenomenon significantly extends the length of the process and decreases the productivity (Gawand 2014; Adnan et al. 2018).

Data from the literature demonstrate that co–fermentation of glucose with glycerol leads to better growth of lactobacilli (El-Ziney et al. 1998). Other studies show specific strains of *E. coli*, *Saccharomyces cerevisiae*, and *Zymomonas mobilis* engineered for simultaneous glucose and xylose utilization via mutagenesis or introduction of a xylose metabolic pathway (Kim et al. 2010). Moreover, glycerol mixed with glucose have been shown to affect the end-product composition. Liu et al. (2010) found that using a glycerol/glucose-based medium for *Propionibacterium acidipropionici* resulted in higher production of propionic acid and decreased byproducts formation (i.e. ethanol).

Co-utilization seemed to be an interesting strategy in fungal cultures. Moon et al. (2004) demonstrated that *Rhizopus* sp. produced relatively low amounts of fumaric acid with a glycerol and rice bran substrate. Interestingly, *Rhizopus arrhizus* cultures on mixtures of crude glycerol and glucose, enhanced fumaric acid biosynthesis (Zhou et al. 2014). However, to our knowledge, the influence of glycerol on the consumption of specific sugars by *Rhizopus* sp. in the production of fumaric acid has not been specifically addressed. This work concerns the fermentative production of fumaric acid from glycerol and a mixture of glycerol with different hexoses or pentoses. For comparative purposes, fermentation experiments with media containing glycerol or sugars as sole carbon sources were also performed.

## Materials and methods

### Microorganisms

*Rhizopus oryzae* strains used in this study were obtained from the NRRL ARS Culture Collection (Peoria, IL USA), NITE Biological Research Center (Chiba, Japan), Deutsche Sammlung von Mikroorganismen und Zellkulturen GmbH (Braunschweig, Germany) and Institute of Agricultural and Food Biotechnology (Warsaw, Poland). *Rhizopus* sp. 29, 30 and 60 were isolated from cereal grains harvested in Lublin Province. All studied fungi were grown on potato dextrose agar (Difco) slants at 30 °C for 4 days and stored at 4 °C. To prepare inocula, agar plates containing sporulated fungi were washed with sterile water to obtain a spore suspension, adjusted to a concentration of 10^5^ spores/ml.

### Medium

The culture medium contained: carbohydrates 40 g/l or carbohydrates 40 g/l and glycerol 20 g/l, KH_2_PO_4_ g/l, MgSO_4 _× 7H_2_O 0.5 g/l, (NH_4_)_2_SO_4_ 1.4 g/l, CaCl_2_ 0.3 g/l and 0.5 ml of microelements solution (FeSO_4 _× 7 H_2_O 5 g/l, MnSO_4 _× H_2_O 1.96 g/l, ZnSO_4_ 1.66 g/l) and distilled water added to make a final volume of 1 l. All media were sterilized by autoclaving at 121 °C. Carbohydrates (glucose, fructose, galactose, mannose, xylose, arabinose, and rhamnose) were prepared and sterilized separately, then added to the medium. 1 g of CaCO_3_ was added to glass tubes and sterilized in a drying oven at 150 °C for 3 h (to avoid contamination when added separately to each flask).

### Culture conditions

10 ml spores were inoculated into 100-ml Erlenmeyer flasks containing 40 ml of culture medium. Cultivation was performed at 35 °C, 200 rpm in rotary shakers (Infors HT, Bottmingen, Switzerland) for 5 days (sole carbohydrates), 7 days (carbohydrates with glycerol) or 8 days (glycerol). 2% (g/v) CaCO_3_ was added to cultures when the pH level was below 4.5 (measured after 24 and 48 h of culture).

### Analytical methods

The final culture broth was acidified with 1.5 M H_2_SO_4_ to neutralize excess CaCO_3_. Next, the post-culture broth was sterilized at 100 °C for 10 min. Then samples were collected and centrifuged at 10,000 rpm for 10 min. The supernatant was analyzed by high performance liquid chromatography (HPLC). Fumaric acid and carbohydrates were determined and quantified by HPLC using Aminex HPX87H organic acid column coupled to UV–Vis diode array (Gilson, Middleton, USA) at 210 nm and a refractive index detector (Knauer, Berlin, Germany). The column was eluted at 65 °C with 5 mM H_2_SO_4_, at a flow rate of 0.5 ml/min. Fumaric acid and carbohydrate standard solutions of known concentrations were used for calibration.

### Calculations

All experiments were performed in triplicate and the means are indicated in the text. The yield of fumaric acid (g/g) is expressed as the amount of product synthesized divided by the amount of carbohydrate consumed by fungi.

## Results

### Fermentation of monosaccharide or glycerol as sole carbon source

Selected *Rhizopus* sp. strains/isolates were screened for their growth on media containing different pentoses/hexoses or glycerol as sole carbon source. The highest production of fumaric acid and the process yield was observed for six strains after culture in the medium containing 40 g/l of xylose (Table 1). The final amount of fumaric acid and process yield were between 18.1 and 19.8 g/l and 0.45–0.50 g/g, respectively. Slightly lower values were obtained after culture of the NRRL-6400 strain when d-mannose was the carbon source (15.0 g/l and 0.38 g/g). Compared to d-xylose and d-mannose, fumaric acid production on d-glucose and d-fructose for all studied fungi were significantly lower. Among strains tested on medium containing galactose, markedly higher concentrations of fumaric acid (14.8 g/l) and its yield (0.37 g/g) were found in the filtrate after culture of *R. oryzae* NRRL-1526 (Table 2).

**Table 1:** *Rhizopus oryzae* strains/isolates producing fumaric acid after growth in media with various pentoses (168 h) or glycerol (192 h) as sole carbon sources (35 °C, 200 rpm)

*Rhizopus* sp. strain/isolate	Substrate							
	Xylose		Arabinose		Rhamnose		Glycerol	
	FA (g/l)	Y_fa/s_ (g/g)	FA (g/l)	Y_fa/s_ (g/g)	FA (g/l)	Y_fa/s_ (g/g)	FA (g/l)	Y_fa/s_ (g/g)
DSM 905	7.0	0.18	0.0	–	0.0	–	1.40	0.04
NRRL3563	17.2	0.44	0.0	–	0.0	–	3.24	0.08
NRRL3613	17.8	0.45	0.0	–	0.0	–	4.38	0.11
NRRL6201	5.7	0.14	0.0	–	0.0	–	1.31	0.03
NRRL2005	18.1	0.45	0.0	–	0.0	–	2.20	0.06
NRRL2582	19.8	0.50	0.0	–	0.0	–	4.21	0.11
NRRL1526	15.9	0.40	0.0	–	0.0	–	3.14	0.08
NBRC4756	17.7	0.44	0.0	–	0.0	–	4.24	0.11
NBRC4773	18.7	0.47	0.0	–	0.0	–	3.9	0.10
NBRC4775	18.6	0.47	0.0	–	0.0	–	1.86	0.05
NBRC4776	18.4	0.46	0.0	–	0.0	–	3.77	0.10
NBRC6154	9.3	0.23	0.0	–	0.0	–	0.16	0.00
NRRL6400	18.3	0.46	0.0	–	0.0	–	5.63	0.14
IAFB781	6.2	0.16	0.0	–	0.0	–	6.10	0.15
R-29	2.3	0.06	0.0	–	0.0	–	0.24	0.01
R-30	0.5	0.01	0.0	–	0.0	–	0.34	0.01
R-60	0.1	0.00	0.0	–	0.0	–	0.80	0.02

FA (g/l), fumaric acid concentration in medium after culture; Y_fa/s_ (g/g), g of fumaric acid produced per g of carbon source consumed

**Table 2:** Fumaric acid production from hexoses (each as the sole carbon source) by various strains/isolates of *Rhizopus* sp. after 120 h of growth (35 °C, 200 rpm)

*Rhizopus* sp. strain/isolate	Substrate							
	Glucose		Fructose		Galactose		Mannose	
	FA (g/l)	Y_fa/s_ (g/g)	FA (g/l)	Y_fa/s_ (g/g)	FA (g/l)	Y_fa/s_ (g/g)	FA (g/l)	Y_fa/s_ (g/g)
DSM 905	4.0	0.10	10.3	0.26	7.3	0.18	11.5	0.29
NRRL3563	3.8	0.10	2.4	0.06	3.7	0.09	9.0	0.22
NRRL3613	3.8	0.10	5.5	0.14	4.2	0.11	7.0	0.17
NRRL6201	4.0	0.10	3.3	0.08	5.0	0.13	14.0	0.35
NRRL2005	3.2	0.08	4.3	0.11	4.6	0.12	7.5	0.19
NRRL2582	3.2	0.08	11.8	0.30	5.3	0.13	9.0	0.22
NRRL1526	4.2	0.11	9.5	0.24	14.8	0.37	13.5	0.34
NBRC4756	4.0	0.10	8.9	0.22	5.0	0.13	10.5	0.26
NBRC4773	7.8	0.20	6.2	0.16	5.7	0.14	10.5	0.26
NBRC4775	3.9	0.10	8.3	0.21	7.3	0.18	9.5	0.24
NBRC4776	7.5	0.19	6.3	0.16	6.3	0.16	9.5	0.24
NBRC6154	3.5	0.09	8.3	0.21	8.2	0.21	12.0	0.30
NRRL6400	6.2	0.16	9.5	0.24	9.2	0.23	15.0	0.38
IAFB781	4.6	0.12	4.5	0.11	6.74	0.17	12.0	0.30
R-29	2.0	0.05	0.02	0.01	7.3	0.18	1.8	0.05
R-30	2.1	0.05	2.9	0.07	–	–	1.6	0.04
R-60	3.1	0.08	0.7	0.02	–	–	1.2	0.03

FA (g/l), fumaric acid concentration in medium after culture; Y_fa/s_ (g/g), g of fumaric acid produced per g of carbon source consumed

*Rhizopus* sp. could effectively utilize glycerol as the sole carbon source in the presence of xylose, but fumaric acid production was low. There was no significant growth of selected strains/isolates in media with arabinose and rhamnose. Thus, fumaric acid production was not observed (Table 1).

### Co-utilization strategy

The most favorable results in the production of fumaric acid by the test strains were obtained after culture on the medium with xylose and glycerol (Table 3). In the latter case, the production of fumaric acid was several times higher than after xylose alone as the sole carbon source. For several strains, these yields were close to 0.90 g/g and were the highest compared to other saccharides. The degree of xylose utilization was generally high, but for most strains, the use of xylose was incomplete, while no significant use of glycerol was observed, or even its minimal synthesis occurred despite the fact that evaporation was taken into account.

**Table 3:** Fumaric acid production from glycerol and xylose mixture by various strains of *Rhizopus* sp. after 120 h of growth (35 °C, 200 rpm)

*Rhizopus* sp. strain	Xylose		Glycerol		Fumaric acid	
	X (g/l)	CV (%)	Gl (g/l)	CV (%)	FA (g/l)	Y_fa/s_ (g/g)
DSM 905	15.24	61.9	22.41	− 12.1	17.74	0.72
NRRL1526	12.18	69.6	24.69	− 23.5	17.76	0.64
NBRC4773	12.98	67.6	21.48	− 7.4	23.28	0.86
NBRC4775	9.12	77.2	20.97	− 4.9	27.82	0.90
NRRL6400	3.93	90.2	20.09	− 0.45	27.82	0.77
IAFB781	0.00	100.0	19.53	2.4	27.92	0.70

FA (g/l), fumaric acid concentration in medium after culture; X (g/l), xylose concentration in medium after culture; Gl (g/l), glycerol concentration in medium after culture; CV (%), consume value; Y_fa/s_ (g/g), g of fumaric acid produced per g of carbon source consumed

Table 4 shows the results of the measurement of fumaric acid concentrations after 7 days of culture of selected *Rhizopus oryzae* strains on the medium with glucose and glycerol. Full utilization of glucose and relatively low of glycerol was found, whereas low synthesis of glycerol was observed for *R. oryzae* IAFB781 strain. For most strains, the production of fumaric acid was higher when glycerol was added to the medium in addition to glucose.

**Table 4:** Fumaric acid production from glycerol to glucose by various strains of *Rhizopus* sp. after 168 h of growth (35 °C, 200 rpm)

*Rhizopus* sp. strain	Glucose		Glycerol		Fumaric acid	
	G (g/l)	CV (%)	Gl (g/l)	CV (%)	FA (g/l)	Y_fa/s_ (g/g)
DSM 905	0.0	100	16.4	18.0	2.92	0.07
NRRL1526	0.0	100	12.5	37.5	7.69	0.16
NBRC4773	0.0	100	16.5	17.5	6.79	0.16
NBRC4775	0.0	100	17.7	11.5	7.77	0.19
NRRL6400	0.0	100	13.1	34.5	8.17	0.17
IAFB781	0.0	100	22.4	− 12.0	8.44	0.22

FA (g/l), fumaric acid concentration in medium after culture; G (g/l); glucose concentration in medium after culture; Gl (g/l), glycerol concentration in medium after culture; CV (%), consume value; Y_fa/s_ (g/g), g of fumaric acid produced per g of carbon source consumed

Unequivocally higher concentrations of fumaric acid and its yields were found when fructose and glycerol were co–utilized (Table 5). In this case, up to 51% efficiency was achieved, while in fructose cultures the highest efficiency was about 30%. In addition, the utilization rate of fructose was close to 100%, while the utilization of glycerol varied, but was higher than glucose–glycerol system and in one case even exceeded 53%.

**Table 5:** Fumaric acid production from glycerol to fructose by various strains of *Rhizopus* sp. after 168 h of growth (35 °C, 200 rpm)

*Rhizopus* sp. strain	Fructose		Glycerol		Fumaric acid	
	F (g/l)	CV (%)	Gl (g/l)	CV (%)	FA (g/l)	Y_fa/s_ (g/g)
DSM 905	1.56	96.2	12.2	37.0	17.2	0.37
NRRL1526	0.54	98.7	14.9	25.5	22.86	0.51
NBRC4773	0.77	98.1	11.1	44.5	17.86	0.37
NBRC4775	0.69	98.3	10.2	49.0	14.23	0.29
NRRL6400	0.96	97.6	9.4	53.0	18.57	0.37
IAFB781	0.54	98.7	13.5	32.5	23.23	0.50

FA (g/l), fumaric acid concentration in medium after culture; F (g/l); fructose concentration in medium after culture; Gl (g/l), glycerol concentration in medium after culture; CV (%), consume value; Y_fa/s_ (g/g), g of fumaric acid produced per g of carbon source consumed

A similar tendency was observed in the culture of selected *Rhizopus* strains on medium with galactose and glycerol, compared to the culture on medium containing galactose as the sole carbon source (Table 6). In this case, the yields of fumaric acid were slightly lower than those after culture in the medium with fructose and glycerol; moreover, significantly lower glycerol utilization was observed by the strains DSM 905, NBRC 1526, and NBRC 4773 after 7 days of culture.

**Table 6:** Fumaric acid production from glycerol to galactose by various strains of *Rhizopus* sp. after 168 h of growth (35 °C, 200 rpm)

*Rhizopus* sp. strain	Galactose		Glycerol		Fumaric acid	
	Ga (g/l)	CV (%)	Gl (g/l)	CV (%)	FA (g/l)	Y_fa/s_ (g/g)
DSM 905	0.54	98.65	18.98	5.1	12.5	0.31
NBRC 1526	0.61	98.48	18.91	5.5	16.29	0.40
NBRC 4773	0.90	97.75	18.42	7.9	13.59	0.34
NBRC 4775	0.69	98.28	17.08	14.6	15.79	0.37
NRRL 6400	0.23	99.43	12.02	39.9	17.67	0.37
IAFB781	0.62	98.45	14.08	29.6	10.74	0.23

FA (g/l), fumaric acid concentration in medium after culture; Ga (g/l), galactose concentration in medium after culture; Gl (g/l), glycerol concentration in medium after culture; CV (%), consume value; Y_fa/s_ (g/g), g of fumaric acid produced per g of carbon source consumed

Of the tested hexoses, the best yields of fumaric acid in the cultures of *Rhizopus* strains studied were obtained when mannose and glycerol were added to the culture medium (Table 7). In this case, more than 50% efficiency was achieved for two strains, while for several others it was close to 50%. After 7-days in culture, the full utilization of mannose and the relatively low utilization of glycerol were found.

**Table 7:** Fumaric acid production from glycerol to mannose by various strains of *Rhizopus* sp. after 168 h of growth (35 °C, 200 rpm)

*Rhizopus* sp. strain	Mannose		Glycerol		Fumaric acid	
	M (g/l)	CV (%)	Gl (g/l)	CV (%)	FA (g/l)	Y_fa/s_ (g/g)
DSM 905	0.0	100.0	14.30	28.5	9.87	0.22
NRRL1526	0.0	100.0	15.67	21.7	11.17	0.25
NBRC4773	0.0	100.0	17.73	11.4	17.67	0.42
NBRC4775	0.0	100.0	14.12	29.4	14.93	0.33
NRRL6400	0.0	100.0	11.03	44.9	21.37	0.43
IAFB781	0.0	100.0	11.66	41.7	12.86	0.27

FA (g/l), fumaric acid concentration in medium after culture; M (g/l), mannose concentration in medium after culture; Gl (g/l), glycerol concentration in medium after culture; CV (%), consume value; Y_fa/s_ (g/g), g of fumaric acid produced per g of carbon source consumed

## Discussion

Different carbon sources had a significant impact on fumaric acid production by *Rhizopus* strains. For most studied strains, glucose was the poorest source of carbon for fumaric acid production compared to fructose and galactose. Moon et al. (2004) examined the effect of different carbon sources on fumaric acid production by *Rhizopus* sp. The highest concentration of fumaric acid was found in the filtrates after *Rhizopus* sp. culture in the presence of rice bran with the addition of glucose and fructose, followed by maltose, starch and galactose, and lastly, glycerol and lactose. In contrast, no fumaric acid was found in post-culture filtrates when sucrose was used as the carbon source. Hemicellulose is one of the most readily available renewable resources that can be hydrolyzed to produce a mixture of monosaccharides containing xylose, arabinose, and rhamnose. There have been several reports on xylose utilization by *Rhizopus* sp. for fumaric acid production (Kautola and Linko 1989; Liu et al. 2015). Kautola and Linko (1989) demonstrated that the highest fumaric acid concentration reached with immobilized cells was 16.4 g/l at 10% initial xylose and a residence time of 10.25 days. In this study, titers obtained on xylose for over half of studied strains were higher, and the culture duration was shorter than previously stated by Kautola and Linko (1989).

Thus far, only a few works have been published on the production of fumaric acid by *Rhizopus oryzae* strains on glycerol as the sole carbon source (Kordowska-Wiater et al. 2012; Zhou et al. 2014; Huang et al. 2015). These studies reported that the production of fumaric acid from glycerol was relatively slow compared to glucose and other saccharides, and the process presented a low efficiency. Huang et al. (2015) obtained 14.9 g/l of fumaric acid with a process yield of 0.248 g/g after G80 strain culture on the medium with crude glycerol. The results presented in Table 1 confirm all those data. In turn, the data presented in Tables 1, 2 show that the type of saccharide is important for obtaining the specific yields of fumaric acid from saccharides. Moreover, they indicate that glucose, used so far as the only source of carbon in fumaric acid production, is not the best carbon source for this process. Zhou et al. (2014) used 80 g/l crude glycerol as a carbon source and obtained 4.37 g/l of fumaric acid after 192 h of culture. After co-fermentation of crude glycerol (40.0 g/l) and glucose (40.0 g/l), they obtained a post-culture filtrate containing 22.81 g/l fumaric acid with an efficiency of 0.346, while a similar yield of 0.35 g/g was obtained after culture on a medium containing 80 g/l glucose using a high-yielding *R. arrhizus* mutant, RH-07-13. Using glycerol to partly replace more expensive pure glucose is an economical approach. These data also suggest the beneficial effect of co-fermentation on the yield of fumaric acid, although they differ significantly from those revealed in this study. The reason may be using of non-genetically modified strains in the own study, unlike the mutants of the *R. arrhizus* strain RH-07-13. The microbial production of fumaric acid by *Rhizopus* sp. was studied to demonstrate the feasibility of monosaccharide utilization with glycerol as a co-existing substrate. The addition of glycerol to the culture medium containing monosaccharides significantly increased the efficiency of fumaric acid production by the *Rhizopus oryzae* strains tested. A several-fold increase in the yield of fumaric acid (more than four times) was obtained in the culture of *Rhizopus oryzae* strains with xylose and glycerol co-fermentation compared to the culture with xylose alone. Moreover, in the presence of fructose, higher glycerol utilization was observed than for other saccharides after the culture of the majority of *Rhizopus oryzae* strains.

It is possible that glycerol as a co-substrate may also play a protective role under stress conditions caused by high initial concentrations of the substrates and/or product. This mechanism is common amongst fungi. Counteracting the stress caused by dehydration consists of polyols accumulation, primarily glycerol, in the outer layers of fungal cell membranes, in order to protect the interior from environmental factors that can stun the action of essential enzymes (Blomberg and Adler 1992). Moreover, fungi are known to produce glycerol, which can accumulate in the cell. Some results from this study indicate production of glycerol after co-utilization with some substrates (xylose and glucose), despite the fact that the evaporation coefficient was taken into account in the calculations. It may be correlated with the aforementioned protective mechanism and occur due to specific substrates and its concentration even when glycerol is present in the fermentation medium.

Effective glycerol utilization observed in some cases of the co-fermentation strategy in this study can be explained by the fact that the fungus has time to adapt its metabolism to consume a second, more complicated, source of carbon like glycerol is. The acceleration of monosaccharide consumption, even without the utilization of glycerol from the co-fermentation media, seems to be a very positive aspect of the strategy suggested in this paper. Although the transportation mechanism of glycerol molecules into fungal cells had already been described (Fakas et al. 2009; Klein et al. 2017), as well as glucose-xylose co-utilization by *Rhizopus* (Liu et al. 2017), an in-depth analysis of the processes in the presence of monosaccharides-glycerol mixtures should be made for a better understanding of the entire mechanism. This work may be a good start to advanced research on *Rhizopus* species metabolism under specific co-utilization conditions. Promising results of fumaric acid production give an opportunity for microbiological production to assist or even replace the chemical synthesis of this acid.
